# “All Hands on Deck”: Psychological Characteristics of Women with Experience of Oncological Disease Participating in Sailing Cruise—A Pilot Study

**DOI:** 10.3390/ijerph192013133

**Published:** 2022-10-12

**Authors:** Julia Wyszomirska, Monika Bąk-Sosnowska, Adriana Modrzejewska

**Affiliations:** 1 Department of Psychology, Chair of Social Sciences and Humanities, School of Health Sciences in Katowice, Medical University of Silesia in Katowice, 40-752 Katowice, Poland; 2 Department of Health Sciences, WSB University, 41-300 Dąbrowa Górnicza, Poland

**Keywords:** sailing, cancer survivors, women, breast cancer, personality, big five, time perspective theory, psychological well-being, health locus of control

## Abstract

Background: In addition to searching for effective methods of treatment, interventions are sought to support well-being, quality of life, mental health, and recovery. Sailing has its specific features, including task orientation, challenges, contact with people, and nature. This specificity may be treated as a potential therapeutic factor, but it is also likely that people with certain psychological characteristics are involved in it. Therefore, the study aimed to assess some psychological features of women with cancer experience who decided to take part in the Onco-Cruise (Polish: Onko-Rejs). Methods: Participants were 56 women (M = 46.73; SD = 9.21). We used NEO-FFI, the Zimbardo Time Perspective Inventory, and The Multidimensional Health Locus of Control Scale. Results: Onco-Cruises participants were characterized by a high level of extraversion (M = 32.48; SD = 7.02; sten score M = 7.21; Mo = 7), openness (M = 31.50; SD = 6.31; sten score M = 7.41; Mo = 8), low neuroticism (M = 21.62; SD = 9.33; sten score M = 4.96), predominance of present hedonistic (M = 12.55, SD = 1.46) and future time perspective (M = 11.39; SD = 2.67), and the internal health locus of control (M = 23.25, SD = 5.43). Conclusion: Group sailing can be favorable for broadly understood health and cancer recovery, but people who choose this activity have certain psychological predispositions, especially indicating high needs for stimulation. Permanent features should be taken into account when proposing various interventions for oncology patients to best suit them to their natural possibilities and preferences and, thus, make them most effective.

## 1. Introduction

According to reports by the World Health Organization, in 2020, one in six people in the world died of cancer, which was a total of 10 million people [1]. Breast cancer is dominant in the female population. Both the majority of cases and deaths occur in women between the ages of 50 and 70, which accounts for approximately 50% of all cases [2]. In Poland, in the last 30 years, the incidence in premenopausal women (20–49 years) has almost doubled, with the annual survival in this group of patients estimated at 94.4%, and the 5-year survival at 78.5% [3]. 

Depending on the characteristics of the cancer and its stage, specific therapeutic methods are used, including cytoreductive surgery, radiation treatment, targeted endocrine/molecular therapy, and chemotherapy [4]. Aggressive treatment causes a number of troublesome side-effects that may persist over the long term. These include: fatigue, headaches, pain and numbness in the limbs (peripheral neuropathy), dental problems, lymphoedema, musculoskeletal symptoms, loss of bone density and osteoporosis, heart problems, new tumors, cataracts, blood clots, amenorrhea, symptoms menopause, sexual problems, infertility, and symptoms related to memory loss and cognitive functions [5]. Cancer and its treatment are significant burdens not only for the sick body, but also for its psyche. Already receiving a diagnosis contributes to the deterioration of the mental state. As shown by a meta-analysis of 39 quantitative studies, following the diagnosis of breast cancer, the incidence of clinically significant symptoms of distress was 39%, anxiety 34%, post-traumatic stress disorder 31%, and depression 20% [6]. Acceptance of the neoplastic disease and active methods of coping with the disease positively affect the quality of life perceived by patients [7], who experience a lower intensity of symptoms related to the disease and its treatment [8]. In turn, passive coping strategies are associated with higher levels of mental stress, cortisol, and tumor necrosis factor alpha, such as more avoidance, negative self-targeting, and less positive reappraisal and focusing on a problem’s solution [9].

One of the commonly used methods of actively coping with stress is physical activity [10]. Scientific reports indicate a beneficial effect of sport also in clinical groups, both among patients with psychiatric [11,12,13] and somatic diseases [14,15]. In the case of cancer patients, there are no meta-analyses that would allow for such a broad extrapolation of results. Research seems to concern more specific methods, a type of sport or a specific group of patients. This is understandable and even recommended taking into account the variety of symptoms, the course of the disease, and the treatment of oncological patients [16]. For example, the potential effectiveness of postoperative exercises in patients with lung cancer [17] or in patients with advanced cancer [16] has been demonstrated, and participation in group Qigong or Tai Chi classes is associated with improvement of the somatic and psychosocial status of cancer patients [18,19]. In turn, participation in dragon boat racing reduces the level of fatigue [20] and positively affects the quality of life of breast cancer patients [21].

Regarding sailing, our information shows that there is currently one pilot study available for cancer patients, involving 19 breast cancer women. The results show a reduction in the level of anxiety and distress and an improvement in the quality of life in the study group. As part of the intervention, in addition to sailing theory and practice, the patients had sessions with a psychologist and a physical exercise specialist [22]. In Poland, since 2015 (with a break during 2020 because of the SARS-CoV-2 pandemic), annual sea cruises for cancer patients—Onco-Cruises (Polish: Onko-Rejs)—have been organized by the Onco-Cruise-I Choose Life Foundation. The Foundation was established to, inter alia, initiate and support innovative solutions in healthcare, and popularize preventive examinations and knowledge about chronic diseases, especially cancer. From 2019, cruises take place in the Mediterranean Sea on the Sail Training Ship (STS) “Fryderyk Chopin”. Its crew consists of a maximum of 53 people, including 7–8 of the permanent crew [23]. Participation is payable, but the costs are at least partially covered by fundraising activities conducted by the Foundation [24]. Due to the characteristics of the crew, the bottom-up nature of the initiative, and the rank of a sailing ship, it is a unique event on a global scale. Contrary to individual clinician-supervised interventions aimed at assessing the effectiveness of sailing for mental health and quality of life conducted in clinical groups [22,25,26], this is an initiative of the patients themselves. The main purposes of the cruises are: to allow people that experienced traumatic experiences to forget about their illness for a moment and feel the real freedom that the sea gives; to break stereotypes about cancer patients by showing that you can live actively, overcome your own weaknesses, do things that are not easy even for a completely healthy person, and enjoy every day with the disease; to show that anyone may develop cancer and to emphasize the importance of preventive examinations that can save lives [24]. There are no additional planned psychotherapeutic, physiotherapeutic, or other interventions. Onco-Cruises participants are a full-fledged crew and perform all work related to the proper functioning of the sailing ship, included in the regulations. There is a 24/7, uninterrupted readiness to work on board. In the three-wing mode, the crew performs navigational watches, galley, port, and anchor watches. Participants steer, work on sails, take part in port maneuvers, perform boatswain’s work, and clean. They are obliged to read and apply the safety rules and to know the running rigging [23].

Sailing, apart from sports and the recreational aspect, is closely related to nature. It helps build a sense of security, relieves anxiety related to diagnosis and treatment, and promotes self-reflection, including understanding life changes [27]. In addition, sailing is characterized by a specificity that should potentially have a positive effect on the mental state and attitude to treating patients, e.g., physical activity, contact with a group, achieving a specific goal, acting for a larger community, the need to overcome difficulties, contact with nature, and the pleasure of sailing. On the other hand, it is associated with similar, although perhaps less severe, stressors experienced by seafarers, e.g., being constantly in a small area with a group of people, separation from loved ones, sea sickness, change in circadian rhythm or sleep deprivation, sometimes long or hard work, exercise, comfort limitation, environmental stressors [28]. Tolerance for the above-mentioned factors, their potential developmental, or negative considerations depends, among others, on mental predispositions.

According to the Big Five Theory, the universal structure of personality consists of the following features [29]: extraversion (related to the amount and quality of social interactions as well as the level of activity and the ability to feel positive emotions), agreeableness (related to attitudes toward other people), conscientiousness (determining the degree of organization, persistence, and motivation in goal-oriented activities), neuroticism (corresponding to adaptation vs. emotional imbalance, associated with the susceptibility to experiencing negative emotions and stress), and openness (determining the individual’s tendency to seek impressions and experiences, cognitive curiosity, and tolerance toward novelty). The listed personality traits are helpful in the analysis of the functioning of people participating in a manned sailing cruise, where they are subjected to intense environmental and interpersonal stimuli. Research indicates that among women with breast cancer, some of the features mentioned especially have an impact on the quality of life in the physical (neuroticism and agreeableness) and mental (neuroticism and conscientiousness) dimensions [30]. The perception of time may be important for the very decision to participate in the cruise, as well as for the behavior and interactions with other people during the cruise. Time Perspective Theory (TPT) [31] explains how a person responds to time and distinguishes five possibilities in this respect: past negative (concentration on unpleasant memories and remembering failures), past positive (returning to positive events, emotions, attachment to tradition, and close relationships), present hedonistic (living in the present moment, seeking pleasure, and impulsive activities), present fatalistic (feeling of having no influence on one’s fate and succumbing to the course of events), and future (anticipation and planning, and active pursuit of goals). The disease and its type can significantly affect the time orientation of patients, as shown in a study comparing healthy, diabetic, and cancer patients [32]. Other studies have shown that the use of time perspective therapy in women with breast cancer significantly reduces the symptoms of post-traumatic stress, anxiety, and depression [33]. In the case of chronically ill people, another characteristic of life decision-making and overall functioning is the health locus of control (HLC). According to Wallston’s concept [34], a person perceives their health as: dependent on their own actions (Internal HLC) and on the influence of other people, especially authorities such as doctors (Powerful Others HLC), or the result of chance (Chance HLC). Regarding cancer patients, HLC may have a significant impact on the effectiveness of treatment, because it is associated with adherence to therapeutic recommendations [35].

Published studies on the health benefits of sailing interventions focus on the clinical aspects of participants [22,25,26,36]. Still, little is known about the nonclinical features that predispose humans, including patients, to choose and/or benefit from this form of activity. The available research results concerning the psychological characteristics of sailors are focused on professional seafarers. For example, in studies involving 300 Polish seafarers, an above-average level of conscientiousness, extraversion, agreeableness, and openness was noted, with low neuroticism at the same time. The strength of stimulation and inhibition processes was normal in them [37]. These issues are of particular interest in the context of the burden of oncological disease and the demands of seafaring. For this reason, the aim of our study was to describe the psychological characteristics of women diagnosed with oncological disease (mainly breast cancer) who participated in Onco-Cruises. We treated it as a pilot study to describe the relatively constant psychological characteristics of the subjects, such as personality structure, time perspective, and the HLC. We predicted that among participants, women would dominate with a high level of extraversion, agreeableness, conscientiousness, openness, but not neuroticism. Moreover, they would be future-orientated, but at the same time know how to enjoy the present and be convinced of the possibility of influencing their health and well-being. We plan to continue the study in the future, analyzing changes in the modifiable psychological characteristics of the participants, which can potentially change as a result of active participation in Onco-Cruises. Currently, however, we want to know what psychological features predispose people to participate in such events and who can potentially benefit from them.

## 2. Materials and Methods

### 2.1. Participants

Women who qualified to participate in a week-long sailing cruise on the Mediterranean, organized by the Onco-Cruise Foundation-I Choose Life, were invited to participate in the study. The inclusion criteria were: consent to participate in the study, age from 18 years, female gender, diagnosis of oncological disease (current or in the past), oncological treatment (current or in the past), and qualification for participation in an Onco-Cruise. The exclusion criteria were: withdrawal of consent to participate in the study. Initial consent to participate in the study was expressed by 102 persons, to whom research questionnaires were sent. A total of 56 sets of questionnaires were received in return, all of which could be included into the analysis.

### 2.2. Methods

The study was carried out by the method of a diagnostic survey, with the use of four questionnaires. Three of them were standardized tools:

NEO-FFI. The Personality Inventory by Paul T. Costa Jr. and Robert R. McCrae in the Polish adaptation of Zawadzki et al. [38] was used to assess the intensity of personality traits in the Big Five model. The questionnaire consists of 60 items making up 5 scales: Neuroticism, Extraversion, Openness, Agreeableness, and Conscientiousness. The respondent, on a 5-point scale, assesses to what extent a given statement is true in relation to them. The tool has sten scores for people aged 15–80, separate for women and men. The measurement reliability estimated with the Cronbach’s alpha coefficient was the highest for the scale: Conscientiousness α = 0.85; Neuroticism α = 0.84; Extraversion α = 0.79; Agreeableness α = 0.69; Openness α = 0.65.

The Polish Short Version of Zimbardo Time Perspective Inventory, PS-ZTPI [39], was used to assess the individual time perspective. The tool is self-reported and consists of 15 items, which are statements that require the respondent to respond on a 5-point Likert scale from “I completely disagree” to “I completely agree”. The items are composed of 5 scales: Past positive (PP), Past negative (PN), Present hedonistic (PH), Present fatalistic (PF), and Future (F). The theoretical basis of the tool is Time Perspective Theory [31]. The reliability in the present study was as follows: PN α = 0.81; F α = 0.7717; PF α = 0.66; PP α = 0.66; for the PH scale, it was low α = 0.21.

Multidimensional Health Locus of Control Scale (MHLC) by Kenneth A. Wellston, Mitchell J. Stein, and Craig A. Smith [34] in Polish adaptation [40]. The MHLC scale includes 18 statements and captures beliefs about generalized expectations in three dimensions of the location of health control: Internal (I)—control over my own health depends on me; Powerful Others (PO)—my own health is the result of the influence of others, especially medical personnel; Chance (Ch)—health is determined by chance or other external factors. The respondent expresses their attitude to the presented statements. The range of results for each of the scales ranges from 6 to 36 points. The higher the score, the stronger the belief that a given factor has an impact on health. In the present study, the highest internal consistency was characterized by the Internal α = 0.76 scale; then, Chance α = 0.66 and Powerful Others α = 0.63. 

We also used our own questionnaire, consisting of 28 questions divided into three parts. The first part included questions about age, education, occupation and current professional activity, place of residence, relationship, and attitude to faith/religion. The next one contains questions about the health status: year of cancer diagnosis, type of cancer(s), number of cancer diseases diagnosed so far, type of cancer treatment (to date, current and planned), chronic diseases, mental disorders, medications taken on a regular basis, and assessment of the frequency and satisfaction with social relations. In the third part, there were questions related to sailing and concerned: past sailing experiences, including participation in previous editions of Onco-Cruises, sailing certification, reasons for the decision to participate in an Onco-Cruise, and expected results.

### 2.3. Study Organization

The data were collected in two periods, before the Onco-Cruise in 2019 and 2021. The declared Onco-Cruise participants were invited and informed about the possibility of taking part in the study through the Onco-Cruise-I Choose Life Foundation. People who gave their informed consent to participate in the study received a complete set of questionnaires. Anonymous questionnaires were filled in by themselves, with unlimited response time, and then returned via the Foundation. The respondents’ answers were placed in a database and then analyzed using STATISTICA 14.0 software. At this stage, we did not have a control group, but we wanted to capture not only the average intensity of the studied features, but above all, their most common prevalence and the overall specificity of the mental functioning of the surveyed women. In such a situation, we believed that a useful approach would be to compare the results to the sten scores in the case of research tools with Polish standardization (NEO-FFI) and the results of Polish adaptations (MHLC, ZTPI), and, in the case of ZTPI, to compare the profile with a Balanced Time Perspective (BTP), i.e., optimal distribution of the intensity of individual traits [41].

## 3. Results

The study group consisted of 56 women aged 25 to 72 years (M = 46.73; SD = 9.21). The vast majority lived in the city (82.14%), had higher education (76.78%), was currently professionally active (71.42%), and was in a relationship (71.42%). Details are presented in Table 1.

The diagnosis of breast cancer dominated (62.50%) in the study group. Most of the respondents have so far been treated with several methods, most often surgical (89.28%) and chemotherapy (69.64%). Almost half of the respondents did not currently have any therapeutic recommendations, and only control examinations (48.21%). This procedure was also recommended for the majority in the near future (62.50%). Some of the respondents declared the presence of a chronic disease other than cancer. Arterial hypertension (25%) and depressive disorders (23.21%) were the most frequently mentioned. The respondents also declared taking medications on a permanent basis, most often Tamoxifen (23.21%) and Levothyroxine (21.42%). Details of the clinical characteristics are presented in Table 2.

The descriptive statistics of the analyzed psychological variables are presented in Table 3.

**Figure 1:**
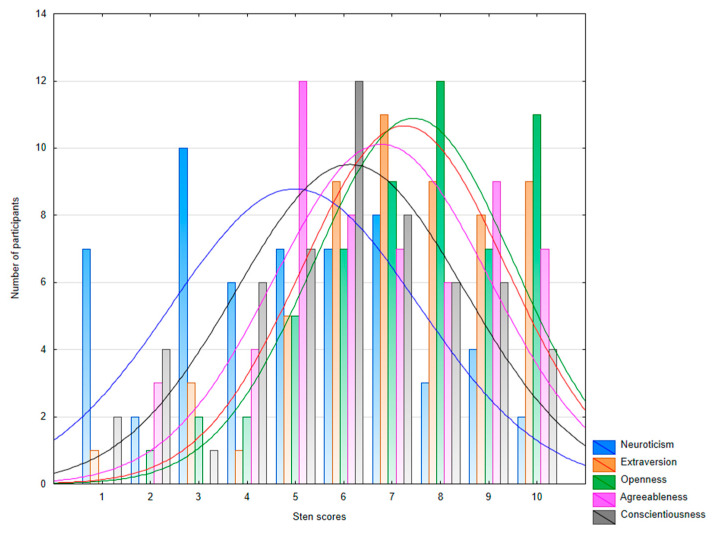
Distribution of NEO-FFI in sten scores.

Figure 1 presents the frequency of results in sten scores. A more detailed analysis of the results on the sten scores indicates their shift toward the high end for openness (M = 7.41; Me = 8; Mo = 8) and extraversion (M = 7.21; Me = 7; Mo = 7), and slightly less pronounced for agreeableness (M = 6.75; Me = 7; Mo = 5) and conscientiousness (M = 6.10; Me = 6; Mo = 6). On the other hand, in terms of neuroticism, the results on the sten scores are shifted toward the low end (M = 4.96; Me = 5; Mo = 3).

**Figure 2:**
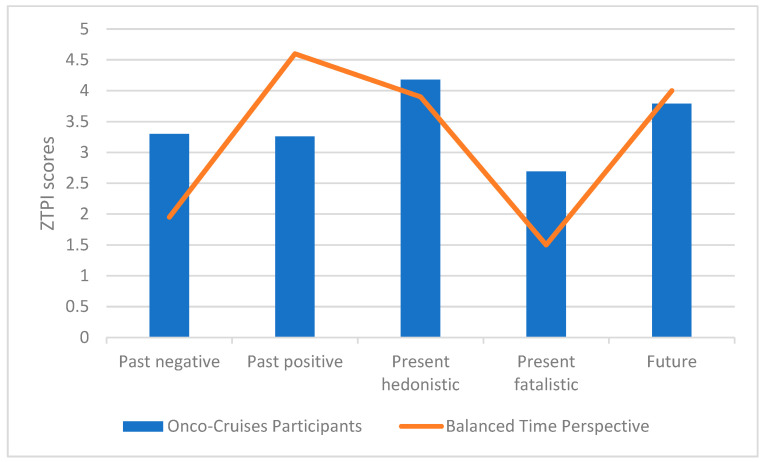
Comparison of Onco-Cruise participant ZTPI scores to Balanced Time Perspective.

Dividing of person’s scores obtained in each scale by 3 [41,42] made it possible to compare their results with BTP (Figure 2). Closest to the theoretical ideal time perspective are the results in the future (M = 3.79 vs. M = 4.00 for BTP) and the present hedonistic scales (M = 4.18 vs. M = 3.90 for BTP), while the greatest deviation was noted in the past negative (M = 3.30 vs. M = 1.95 for BTP) and the past positive scales (M = 3.26 vs. M = 4.60 for BTP).

## 4. Discussion

In our study, we focused on describing the relatively constant psychological characteristics such as personality, temporal orientation, and the health locus of control. We assumed that the respondents would be dominated by people with a high level of extraversion, agreeableness, conscientiousness, and openness to experience, but not neuroticism.

The results of our study showed that the level of extraversion of the surveyed women is high, which means they tend to focus their energy, orientation, and attention on the outside world, including social contacts, having fun, and seeking stimulation [38,43]. Research indicates that exactly the extrovert personality can lead to optimistic expectations, and such individuals are also more confident about the benefits of cancer research prophylaxis [44]. Furthermore, an extroverted person may be more likely to seek social support [45] in order to increase their ability to cope with the challenge of the disease in this regard, which may be a factor influencing participation in an Onco-Cruise. 

Another result obtained in our study indicates an average, and most often, low level of neuroticism in the respondents. Previous studies have shown that high levels of neuroticism are associated with negative health behaviors [46], also leading to depression and anxiety symptoms in cancer patients [47]. High levels of neuroticism also lead to less adaptive coping strategies [48]. There are few studies linking extraversion and neuroticism to the mental health in persons with cancer [49,50,51]. One of them indicates that high levels of neuroticism decrease the quality of life and are associated with the intensification of depression, anxiety, and helplessness in patients with breast cancer [49]. A study of 170 patients with different types of cancer showed that low levels of neuroticism and high extraversion are associated with better mental health indicators and appear to be protective factors for mental health in this patient group [50]. Another study showed that in breast cancer patients, the effects of neuroticism and extraversion outweigh the importance of surgical intervention for quality of life [51]. In general, the results in terms of extraversion and neuroticism can be treated as pro-health resources of the surveyed women. It is well known that personality influences human health behavior [52], and in order to adjust the appropriate health intervention to the patient’s unique characteristics, including their personality [53], it is worth looking at slightly less popular traits than extraversion and features of neuroticism. Subsequent results on the constant features obtained in this study revealed a high level of conscientiousness, openness, and agreeableness. People with high conscientiousness may be characterized by a lack of difficulties in the implementation of planned activities, changes in health behaviors, and adherence to doctor’s recommendations [54]. This may confirm the results of our study, taking into account the willingness of the surveyed women to participate in an Onco-Cruise, which is associated with adherence to specific, sometimes uncomfortable, rules and changes in their daily functioning. High agreeableness is a preference for putting forward a good relationship, willingness to support others, and cooperation over the implementation of individual goals. It is also about trusting people [38,43]. Such features may facilitate functioning during the voyage and may foster the creation of a good, supportive atmosphere during the cruise and prevent conflicts. According to the theory of interpersonal relations and cancer [55], the creation of support groups for ill people may have a positive impact on their functioning in the disease.

Agreeableness is most associated with positive interpersonal behavior [56]. Interestingly, the results of previous research indicate that, depending on the level of agreeableness, the effect of using coping strategies during cancer and after its cure may differ [57]. Negative implications of high agreeableness include a slightly lower tendency to engage in healthcare decisions, including selecting treatment choices [58]. Therefore, in patients with high agreeableness, it is recommended to appreciate the benefits of the ability to maintain good interpersonal relationships and social support [59,60], and encouraging involvement in health-related behaviors (e.g., a healthier diet and physical activity) can be crucial to the well-being of the survivors. To the best of our knowledge, there are few studies that link openness to cancer experience. Generally, people who are open to experiences tend to discover new things in their lives and they see them as less threatening [61]. We suppose that participation in an Onco-Cruise may provide new experiences related to the perception of the disease and one’s abilities in relation to the disease and may be helpful in getting used to the symptoms and consequences of treatment. However, it is also dictated by factors unrelated to the disease, including pure curiosity and the need to accumulate new experiences, activities, cultures, people, and new competences, i.e., high openness. A holistic view of the personality profile of the surveyed women indicates a high need for stimulation to obtain optimal functioning, in terms of physical factors, intensity of stimuli, as well as social and cognitive ones. Additionally, many of them are characterized by high emotional stability [62]. In general, such a set of features can be treated as a psychological health resource, both in the course of dealing with the disease and treatment, as well as in recovery, activity, and coping with everyday stress. In conclusion, personality trait assessments should be routinely carried out in conjunction with standard clinical assessments as they are an important determinant of tailoring support programs, treatment, and various interventions for people with cancer [63].

When we were designing the study, we assumed that the participants would have both a strong future orientation and a present hedonistic and would be convinced of the possibility of influencing their health and well-being. Indeed, in terms of temporal orientation, the participants of our study obtained the highest results in the present hedonistic and future. However, due to the low reliability of the scale of the present hedonistic, we treat the results with great caution. It is imperative to check them in future research. The combination of a strong present hedonistic and future perspective may seem surprising. Most often, these two features are presented as two poles of involvement in each area of time: involvement in the present steals the layers of attention directed toward the future [64]. However, in the concept of TPT, there are two independent factors [31]. Interestingly, the future perspective can act as a buffer for the negative effects of a strong present hedonistic. The present hedonistic is associated with a tendency to intentionally seek intense stimulation, pleasure, activity that guarantees immediate gratification, and a tendency for risk, which can be partly prevented by a strong tendency to take into account the future consequences of one’s behavior and have distant, precise goals [41]. Undoubtedly, the hedonistic perspective has more connections with poor health, and the future has more positive impact on health behavior and well-being [65,66]. The tendencies and behaviors of the Onco-Cruise participants are probably a result of these seemingly contradictory features. In relation to these two perspectives, such persons represent a risky attitude toward life (present hedonistic), which may translate into the choice of participation in an Onco-Cruise as a risky expedition and the willingness to achieve goals for the award that is the sense of satisfaction (future) [67]. The present hedonistic is also the ability to appreciate the current moments, people, and events and the ability to affirm life [41], which may increase the possibility of using potential health-promoting factors present on the cruise (appreciating contact with nature and people, admiration for beauty of sailing, and, therefore, experiencing positive emotions, etc.). Previous studies, focused on the time perspective of people with cancer, showed that planning the future translates into optimistic behavior in everyday life [63]. It is obvious that the participants’ BTP performance differs from a theoretical ideal model. However, it can be seen that it is precisely in the present hedonistic and future that they are similar to people assessed as BTP [42], and even close to the ideal time perspective [41]. The relatively high level of negative past perspective draws attention. We do not know if it is a feature of the respondents or it was shaped to some extent as a result of the experience of the disease, but regardless of the cause, it is the least desirable feature—the most anti-health one in the profile of the surveyed women. It can be associated with poorer quality of life, well-being, or mood [68,69,70] also in cancer patients [71]. Summing up, we believe that the decision to participate in an Onco-Cruise, the involvement in its planning, and then the intensive use of time on the cruise itself help to look optimistically to the future and satisfy the need for impressions and, at the same time, achieving the goals set.

The last feature analyzed in the study was the health locus of control. We assumed that the respondents would be convinced of the possibility of influencing their health and well-being, despite the fact that their treatment was largely based on the medical intervention. The examined women obtained the highest result in the internal health locus of control. Previous studies have confirmed that the sense of control over their own health in cancer patients is associated with better mental adjustment [72], as well as a lower level of depression [73]. Interestingly, healthy women who believe that their health condition results from their own abilities and behavior more often have high scores in this dimension [74]. It can be assumed that the surveyed women want to have a sense of such control and influence on their own health, and participation in an Onco-Cruise is an expression of this. Similar results on internal control were obtained by Naz et al. [75], who showed that people with breast cancer with greater internal control are more motivated to fight the disease. Comparing our results with the results obtained by Polish women with breast cancer [40], our respondents are characterized by the opposite pattern. The highest result was in terms of the internal locus of control and the lowest was external control and chance, while in the above-mentioned study, external control clearly dominated over internal control [40]. Other studies have also indicated a higher sense of external control among cancer patients, associated with lower internal control [74], which was not confirmed in our study. In addition, other studies conducted especially in the rural population suggested that patients are convinced of the external control of their health and the role of chance [72]. It should be noted that our participants during the study period were in fairly good health. Many did not require further oncological treatment, but only recommended regular checkups. Most of them remained professionally active and, in their subjective opinion, felt strong enough to face the cruise’s challenges. Therefore, the result seems specific not so much for oncological ill women as for sailing women with cancer experience, whom participation in Onco-Cruises allows them to alleviate both physical and mental suffering and helps to maintain and improve their health and mental state. Research confirms that just being at sea has a positive effect on well-being [76], and physical activity also improves it in general [77], while sailing brings many health benefits, both physical and mental [78]. 

Summarizing the results of our study on relatively constant psychological features, such as personality structure, time perspective orientation, and the locus of health control, it can be assumed that they are significant for making a decision about participation in an Onco-Cruise. It can be concluded that in the studied group, many psychological features have pro-health values and may constitute an important element of planning secondary health prophylaxis. Onco-Cruises most likely fulfill their pro-health role as declared by the participants and the Foundation, but it is naturally selected for this type of activity—it is chosen by people with specific psychological characteristics. Most likely, the key may be an individual need for stimulation, manifested inter alia by a high level of extraversion and openness and low neuroticism. However, considering the potential invariability of these features, future research should also take into account more factors that build motivation to participate in such endeavors as the Onco-Cruise. Undoubtedly, the combination of fixed features with factors influencing motivation could allow for more effective psychological help, which is needed in the face of cancer.

There are some limitations to this study. First, it was a pilot study without a control group. Our main goal was the initial understanding of the characteristics of psychological traits, but also the collection of demographic and clinical data, in order to be able to adequately select the control group in the next stages of the project.

A serious limitation is the low reliability of the ZTPI present hedonistic scale obtained in the present study. Similarly, in the Polish adaptation of the tool, this scale obtained the lowest reliability rates α = 0.45 [39]. In our study, one of the items turned out not to correlate with the other two: “I prefer friends who are spontaneous rather than predictable”. We suspect that this may be due to a cultural factor. In Poland, friendship is often a synonym of stability and predictability, which is even reflected in the proverbs, e.g., “A friend in need is a friend indeed” or “Friends for Life, Friends for Death”. Perhaps the item in question resulted in the automatic selection of answers more indicative of a greater preference for predictability than spontaneity in friends, which did not reflect real preferences. Regardless of the reason, this question and the entire scale require re-verification and psychometric evaluation. Although the measurement of the present hedonistic itself may be biased, taking into account the overall profile of the respondents, we suspect that the obtained results actually reflect the great importance of the hedonistic present for the surveyed women.

Due to the lack of standardized norms for MHLC without a control group, it is difficult to interpret the results based solely on measures of central tendency.

## 5. Conclusions

Women experiencing oncological disease who participated in Onco-Cruises were characterized by a high level of extraversion, agreeableness, openness, and conscientiousness, and low level of neuroticism, predominance of a present hedonistic and future perspective over time, and an internal health locus of control. 

The pilot study had several goals. The essential element was the initial recognition of the constant psychological characteristics of women participating in Onco-Cruises. We also wanted to obtain information on the demographic and clinical characteristics of participants. This will enable us to better plan further studies on the impact of participation in Onco-Cruises on the mental and emotional state (anxiety, stress, mood, adaptation to the disease, and examination before and after the cruise). Due to the wide scope and length of future surveys, we wanted to distinguish such constant features that are particularly expressive and can potentially be associated with the reception of the cruise experience in order to control them at a later stage of the research. In the pilot study, we chose well-known and popular psychological tools, which makes it possible to compare our results to other research. We wanted to check if they would be appropriate in the context of the specificity of the group and the nature of the further study. Therefore, taking into account the intensity of individual features, their potential importance for further research in relation to other already available results and the research economy in relation to the psychometric values of the tools, we decided to control time perspective and personality traits in the Big Five concept at a later stage. However, due to the planned extensive scope of the study and individual comments on the length of the current survey, we considered the study with a shorter Big Five measuring tool, e.g., the Polish version of the Ten Item Personality Inventory [79]. We decided not to assess the health locus of control.

The study also allowed us to use current experiences and results for further research in the field of relatively constant psychological traits associated with different forms of activities undertaken by patients with chronic diseases that differ in intensity, duration, and nature. This, in turn, can be used to learn about the preferences of patients and to create various adequate forms of activity proposals, tailored to personal characteristics. Regarding this option, we suggest that it is worth staying with the tools used in the pilot study.

## Figures and Tables

**Table 1:** Demographic characteristics.

Variable	N	%
Dwelling-place		
Village	10	17.85
City with up to 100,000 inhabitants	18	32.14
City with over 100,000 inhabitants	28	50.0
Education		
Primary	0	0
Vocational	4	7.14
Secondary	9	16.07
Higher	43	76.78
Employment		
Currently working	40	71.42
Currently on sick leave/rehabilitation benefit	6	10.71
Pensioner	5	8.92
Retiree	4	7.14
Unemployed	1	1.78
Partner status		
In relationship	40	71.42
Single	16	28.57
Faith		
Practicing believer	24	42.85
Non-practicing believer	21	37.50
Doubter	7	12.50
Non-believer	4	7.14

**Table 2:** Clinical characteristics.

Variable	N	%
Oncological diagnosis		
Breast cancer	35	62.50
Ovarian cancer	7	12.50
Cervical cancer	4	7.14
Hodgkin’s lymphoma	3	5.35
Thyroid cancer	3	5.35
Other	9	16.07
Previous oncological treatment		
No recommendation or only observation	0	0.0
Surgical treatment	50	89.28
Chemotherapy	39	69.64
Radiotherapy	34	60.71
Hormone therapy	26	46.42
Other	10	17.85
Current oncological treatment		
No recommendation or only observation	27	48.21
Surgical treatment	0	0.0
Chemotherapy	4	7.14
Radiotherapy	2	3.57
Hormone therapy	23	41.07
Other	4	7.14
Planned oncological treatment		
No recommendation or only observation	35	62.50
Surgical treatment	3	5.35
Chemotherapy	4	7.14
Radiotherapy	0	0.0
Hormone therapy	15	26.78
Other	0	0.0
Chronic diseases		
Hypertension	14	25.00
Hashimoto disease/hypothyroidism	9	16.07
Diabetes	2	3.57
Asthma	2	3.57
Depressive disorders	13	23.21
Anxiety disorders	13	23.21
Other	5	8.92
Medications treatment		
Tamoxifen	13	23.21
Levothyroxine	12	21.42
Selective serotonin reuptake inhibitors	7	12.50
Bisoprolol	3	5.35
Other	33	58.92

**Table 3:** Descriptive statistics of psychological variables in the study group.

Variable	M	SD	Me	Mo	Min.	Max.
NEO Five-Factor Inventory (NEO-FFI)						
Neuroticism	21.62	9.33	22.00	23.00	2.00	42.00
Extraversion	32.48	7.02	33.00	33.00	14.00	47.00
Openness	31.50	6.31	32.00	33.00	18.00	44.00
Agreeableness	34.53	5.74	35.00	33.00	20.00	48.00
Conscientiousness	34.57	7.82	35.50	35.00	12.00	48.00
Zimbardo Time Perspective Inventory (ZTPI)						
Past negative	9.92	3.49	10.00	13.00	3.00	15.00
Future	11.39	2.67	12.00	12.00	4.00	15.00
Present hedonistic	12.55	1.46	13.00	13.00	9.00	15.00
Present fatalistic	8.08	3.03	8.00	8.00	3.00	15.00
Past positive	9.80	2.95	10.00	10/11/12	3.00	15.00
Multidimensional Health Locus of Control Scale (MHLC)						
Internal	23.25	5.43	24.00	26.00	9.00	34.00
Powerful Others	20.57	5.33	20.00	19.00	9.00	32.00
Chance	20.19	5.76	19.50	18.00	9.00	36.00

## Data Availability

The data presented in this study are available on request from the corresponding author.
